# Unilateral vs. Bilateral Selective Cerebral Perfusion for Acute Type A Aortic Dissection with Frozen Elephant Trunk: Systematic Review and Meta-Analysis

**DOI:** 10.3390/jcm14186392

**Published:** 2025-09-10

**Authors:** Massimo Baudo, Michele D’Alonzo, Claudio Muneretto, Stefano Benussi, Lorenzo Di Bacco, Fabrizio Rosati

**Affiliations:** 1 Department of Cardiac Surgery Research, Lankenau Institute for Medical Research, Main Line Health, Wynnewood, PA 19096, USA; massimo.baudo@icloud.com; 2 Cardiac Surgery Unit, Poliambulanza Foundation Hospital, Via Bissolati 57, 25124 Brescia, Italy; michele.dalonzo@poliambulanza.it; 3 Department of Cardiac Surgery, Spedali Civili di Brescia, University of Brescia, 25123 Brescia, Italy; claudio.muneretto@unibs.it (C.M.); stefano.benussi@unibs.it (S.B.); rosati.fabri@gmail.com (F.R.)

**Keywords:** acute aortic dissection, frozen elephant trunk, selective antegrade cerebral perfusion, total arch replacement, cardiac surgery, meta-analysis

## Abstract

**Background/Objectives**: Previous studies failed to demonstrate the best cerebral perfusion option during total aortic arch replacement surgery. We therefore sought to investigate clinical results of patients who received unilateral (u) versus bilateral (b) selective antegrade cerebral perfusion (SACP) during total aortic arch replacement by means of the frozen elephant trunk (FET) technique for acute type A aortic dissections (ATAADs). **Methods**: A systematic review and meta-analysis was performed by including relevant studies reporting outcomes of patients with ATAADs undergoing total arch replacement with the FET technique using either unilateral or bilateral SACP. Pubmed, ScienceDirect, SciELO, DOAJ, and Cochrane library databases were searched until May 2023. **Results**: A total of 44 papers with 5983 patients were included, 3872 for unilateral SACP and 2111 for bilateral SACP. Although patients undergoing bSACP presented a higher incidence of preoperative comorbidities compared to uSACP, there was not a significant difference in terms of mortality and major postoperative complications between the two groups. Distal body circulatory arrest time was not associated with any outcome at meta-regression, while longer SACP times in the whole population were significantly associated with higher neurological complications. Significant increased mortality was found in long uSACP. **Conclusions**: Our analysis showed that centers already apply the appropriate perfusion strategy tailored to the complexity of the patient’s condition. It is therefore crucial to tailor the approach to the complexities of individual patients rather than seeking a definitive superiority between the two perfusion techniques to optimize outcomes during FET for ATAAD. Further studies are warranted to confirm these results.

## 1. Introduction

Acute type A aortic dissection (ATAAD) is a critical surgical emergency with a substantial risk of mortality [1]. The primary goal of the surgical approach involves the need for the exclusion of the entry tear, located in most cases at the level of the ascending aorta or the inner curvature of the arch. However, the presence of an entry tear into the arch and the extension of the false lumen into the aortic arch and the descending thoracic aorta may necessitate more extensive interventions. Consequently, more comprehensive techniques have been introduced over the years such as total arch replacement (TAR) by means of the frozen elephant trunk (FET) procedure [2]. The device demonstrated excellent performance, particularly regarding ease of implantation, as well as false lumen exclusion and thrombosis; however, this technique was still associated with a not negligeable incidence of major neurological complications [3,4].

While some of these complications seem to be related to the device itself, such as spinal cord ischemia and injury, others seem to be a direct consequence of the cerebral perfusion strategy used during the phase of the device’s positioning and the open distal anastomosis [5]. In fact, different protective cerebral perfusion strategies are available at present, with antegrade or retrograde cerebral perfusion leading to improved postoperative results when compared to deep hypothermic circulatory cerebral arrest [6]. Furthermore, selective antegrade cerebral perfusion (SACP) can be performed either unilaterally (uSACP) or bilaterally (bSACP). The comparison of these two techniques did not demonstrate a clear advantage of one over the other in previous studies [7,8,9], but these results were derived from comparisons involving various aortic pathologies and/or techniques. Even a recent meta-analysis on the general surgical treatment of ATAAD, which suggested a possible advantage of bSACP, included different surgical techniques [10]. Therefore, we sought to investigate clinical outcomes of patients who received uSACP versus bSACP during aortic arch replacement surgery for ATAAD by means of FET procedure.

## 2. Materials and Methods

### 2.1. Protocol and Registration

The protocol for this review is registered with the PROSPERO database of systematic reviews (ID: CRD42023465077). Because the analysis was based exclusively on previously published data and did not involve direct patient participation, institutional ethics approval and informed consent were not necessary. The datasets used and analyzed during the current study can be obtained from the corresponding author upon reasonable request.

### 2.2. Search Strategy

**Figure 1:**
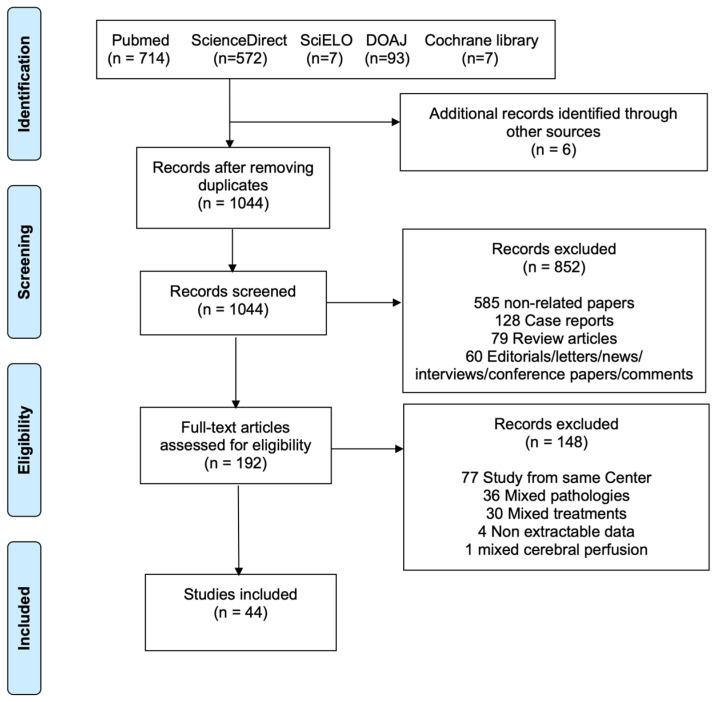
Preferred Reporting Items for Systematic Reviews and Meta-Analyses (PRISMA) flowchart of the included studies.

This review adhered to the Preferred Reporting Items for Systematic Reviews and Meta-Analyses (PRISMA) framework [11]. The study selection process is summarized in the flow diagram, Figure 1. A systematic literature search was performed across PubMed, ScienceDirect, SciELO, DOAJ, and the Cochrane Library up to May 2023, targeting reports of patients undergoing TAR with the FET procedure using either unilateral or bilateral SACP for ATAAD. The complete search strategy is provided in Table S1. To ensure thoroughness, reference lists of eligible papers and prior reviews were also examined (backward snowballing). Screening for eligibility was conducted independently by two investigators (M.B. and M.D.), with any discrepancies resolved through discussion and, when necessary, consultation with a third reviewer (F.R.).

### 2.3. Inclusion and Exclusion Criteria

We included studies that reported outcomes of patients with ATAADs undergoing TAR with the FET technique using either unilateral or bilateral SACP. Bilateral SACP was considered when perfusion was established simultaneously in both the right and left carotid (or their parent vessels), ensuring direct antegrade blood supply to both hemispheres independently, regardless of variations or incomplete collateral circulation. This contrasts with unilateral SACP where perfusion was delivered into one cerebral vessel (commonly the right axillary or innominate artery). Adequacy of perfusion to the contralateral hemisphere depends on the integrity of the circle of Willis and collateral flow pathways. Exclusion criteria included studies involving populations with aneurysms or other non-urgent aortic conditions, as well as those employing surgical methods beyond TAR or techniques other than FET. Additionally, studies incorporating supra-aortic vessel stent branching techniques were omitted. Articles employing cerebral perfusion techniques other than unilateral or bilateral SACP were not taken into account. Papers that did not provide distinguishable data between uSACP and bSACP, or that specified that the strategy could change according to clinical worsening, were also excluded. Studies with fewer than 10 patients, along with case reports, series, reviews, abstracts, presentations, comments, and papers not in English, were also disregarded. In cases of multiple publications from the same institution, the study periods were evaluated, and the study with the largest sample size was included in the event of any time overlap.

### 2.4. Data Extraction

The extraction of data was carried out using Microsoft Office 365 Excel software (Microsoft in Redmond, Washington, DC, USA). The information gathered included details about the study’s timeframe, location, country, and the size of the sample. Specifics about the patients’ characteristics, intraoperative data, and postoperative outcomes were also abstracted.

Given the likelihood of encountering discrepancies in reported information across cases, it was anticipated that each article might introduce unique variables not present in others. Therefore, a degree of interpretation was necessary to address missing data pertaining to certain variables. The denominators for specific variables served as indicators of their respective percentage values. In presenting this data, the denominators were based on explicit mentions of variables’ presence or absence, or on reasonable inferences where applicable.

### 2.5. Critical Appraisal and Outcomes of Interest

The quality of included non-randomized studies was assessed through The Risk of Bias in Non-Randomized Studies of Interventions tool (ROBINS-I) [12].

The primary endpoint was hospital mortality, while secondary endpoints included postoperative cerebrovascular accidents, spinal cord ischemia, bleeding requiring surgery, and need of dialysis.

### 2.6. Statistical Analysis

For postoperative outcomes, pooled event rates (PERs) or means (PEMs) were calculated and were presented with a 95% confidence interval (CI). In all analyses, studies were weighted by the inverse of the variance of the estimate for that study, and between-study variance was estimated with the DerSimonian–Laird method with random effects model. Studies with zero occurrences were included in the meta-analysis, and zero cell frequencies were adjusted using a treatment arm continuity correction. Equivalence hypothesis testing was set at a two-tailed 0.05 significance level. Heterogeneity was assessed using the Cochran Q test, with I^2^ values. Assessment of publication bias utilized funnel plots and Egger’s regression test, where applicable.

Meta-regression was performed to further analyze the relation between the cerebral perfusion strategy with circulatory and SACP times for neurological complications and mortality. Results were expressed as odds ratio (OR), 95%CI, and *p*-value.

All analyses were carried out using R, version 4.3.1 (R Project for Statistical Computing, Vienna, Austria) and RStudio version 2023.06.0+421. The “meta” package was employed for the meta-analysis.

## 3. Results

### 3.1. Study Selection and Characteristics

The systematic review process is outlined in Figure 1. The literature search identified 1393 potentially eligible studies. Six additional articles were identified through backward snowballing. After removal of duplicates, 1044 studies were screened. Among these, 192 full-text articles were assessed for eligibility. Forty-four articles [2,13,14,15,16,17,18,19,20,21,22,23,24,25,26,27,28,29,30,31,32,33,34,35,36,37,38,39,40,41,42,43,44,45,46,47,48,49,50,51,52,53,54] met our inclusion criteria (Supplementary Material), with a total of 5983 patients, 3872 for uSACP and 2111 for bSACP. Publication year ranged from 2008 to 2023, and the sample size ranged from 10 to 1522 patients. Out of 5269 patients with stent information, most (79.7%, n = 4198) received a Cronus (MicroPort Medical, Shanghai, China), 10.5% (n = 551) a J Graft Open Stent/Frozenix (Japan Lifeline Co, Ltd., Tokyo, Japan), 6.5% (n = 343) a Thoraflex Hybrid (Terumo Aortic, Glasgow, Scotland, UK), 3.2% (n = 166) an E-Vita (Artivion, Kennesaw, GA, USA), 0.5% (n = 28) a Gianturco (Microport Medical Corp., Shanghai, China), 0.2% (n = 10) a Sutureless Integrated Stented (SIS) graft (Beijing Percutek Therapeutics Inc., Beijing, China), and 0.1% (n = 4) a Talent (Medtronic Inc., Minneapolis, MN, USA). Most procedures were carried out with separate reimplantation of epiaortic vessels and Zone 2 distal anastomosis. Details of the individual studies are shown in Tables S2 and S3. The studies included 3 propensity-matched studies and 41 observational studies. The critical appraisal of the included studies is displayed in Table S4.

### 3.2. Baseline Patients’ Characteristics

The two groups showed significant differences in terms of preoperative characteristics. In particular, bSACP patients were significantly older (59.8 ± 5.4 vs. 51.6 ± 6.4, *p* < 0.001), reported more previous cerebrovascular events (8.3% vs. 4.3%, *p* < 0.001) and previous cardiac surgery (4.7% vs. 2.5%, *p* = 0.003), and suffered more from COPD (8.7% vs. 1.2%, *p* < 0.001), chronic kidney disease (13.2% vs. 3.0%, *p* < 0.001), and coronary artery disease (13.3% vs. 6.7%, *p* < 0.001), when compared to uSACP. Moreover, patients in the bSACP group had a worse clinical presentation with a higher incidence of acute neurological deficit (9.5% vs. 1.7%, *p* < 0.001), lower hemodynamic performance (18.2% vs. 3.8%, *p* < 0.001), and hemopericardium (17.8% vs. 5.2%, *p* < 0.001), when compared with the uSACP group. Baseline patients’ characteristics are summarized in Table 1.

### 3.3. Meta-Analysis

Intraoperatively, patient with uSACP underwent a significantly higher rate of root replacement surgery (PER: 30.11%, 95%CI: 23.95–37.07, vs. 12.90%, 95%CI: 7.77–20.66, *p* = 0.0012), particularly utilizing the Bentall technique (PER: 28.64%, 95%CI: 22.66–35.49, vs. 8.64%, 95%CI: 5.11–14.24, *p* < 0.0001), with respect to bSACP. The PEM for SACP time exhibited a significant discrepancy between the two groups (bSACP: 80.5 min, 95%CI: 54.7–118.5, vs. uSACP: 49.8 min, 95%CI: 39.0–63.5, *p* = 0.0395). The target temperature was significantly lower in the uSACP compared to bSACP (uSACP: 22.5 °C, 95%CI: 21.5–23.6 vs. bSACP: 26.0 °C, 95%CI: 25.1–26.9, *p* < 0.001). No other significant differences were noted, as indicated in Table 2.

There was no significant difference in mortality and postoperative complications between the unilateral and bilateral SACP groups, Table 3.

### 3.4. Meta-Regression

Distal body circulatory arrest was not associated with any outcome at meta-regression. On the other hand, longer SACP times in the total population were significantly associated with higher neurological complications, and longer SACP times in the uSACP were significantly associated with higher mortality (Table 4).

## 4. Discussion

This meta-analysis showed the following: (1) institutions that utilized bSACP would perform a TAR+FET in patients with more comorbidities than institutions using uSACP, possibly because the institutions utilizing uSACP, in a scenario of higher comorbidities, performed a less extensive operation; (2) the most concerning complications (SCI, CVA, postoperative bleeding, dialysis, and hospital mortality) remained relevant regardless of the cerebral perfusion approach applied; (3) longer SACP times, but not longer distal body circulatory arrest times, were associated with worse outcomes.

The current analysis highlighted a hospital mortality ranging from 8% to 9%, along with a postoperative incidence of SCI at 3–4% and CVA at 6–9%. Nevertheless, it remains a demanding and intricate procedure, particularly in the urgent setting of ATAAD [55,56]. This complexity is reflected in the mortality and the most concerning neurological complications rates. These findings align with a recent meta-analysis that specifically addressed these complications [5]. In line with prior reviews focusing on perfusion strategies across different aortic arch conditions and techniques, our study also revealed no notable distinctions between uSACP and bSACP [7,8,9], thus suggesting FET procedures during ATAAD remain an intricate procedure with a high incidence of mortality and neurological complications. A recent meta-analysis analyzing ATAAD outcomes, including heterogeneous surgical interventions, indicated that uSACP may offer particular benefits in the context of TAR [10]. However, this finding was based on a subgroup analysis that was likely influenced by a selection of studies not specifically designed to address the question in this subset of patients. To our knowledge, we are the first in confirming this relation in the context of TAR with FET for ATAAD.

The comprehensive analysis of critical postoperative outcomes following FET procedures for ATAAD confirmed a very intuitive association. Bilateral perfusion strategies were generally performed in more comorbid patients. Longer SACP times were also required, suggesting surgical teams expected a more challenging intervention in this specific subset of patients. However, postoperative results showed a surprisingly degree of similarity between the two groups. A possible explanation is that institutions employing bSACP may have tended to perform TAR+FET in patients with greater comorbidity burdens and/or more extensive procedures, whereas those using uSACP might have opted for less extensive procedures in similarly high-risk patients, possibly highlighting a different expertise and/or preference. Overall, institutions adopted only a single cerebral perfusion strategy. In other words, this intriguing finding emphasizes the appropriate perfusion strategy should be tailored to the complexity of the patient’s procedure. In this regard, a comprehensive understanding of advantages and disadvantages associated with each method allowed for a technical customization, thus yielding similar surgical results and optimal outcomes for each individual case [57]. For the majority of patients without significant pathologies or less extensive procedures, uSACP seemed to offer adequate safety, while bSACP was better employed for the more comorbid patients with the worst clinical presentation from a neurological point of view and/or more complex procedures [58].

The reported target body temperature differed between unilateral and bilateral SACP. In unilateral perfusion, surgeons often employ deeper systemic hypothermia to safeguard the contralateral hemisphere in case intracranial collaterals are inadequate. Conversely, bilateral perfusion allows direct supply to both hemispheres, making higher target temperatures feasible and thereby shortening cooling and rewarming times, which can reduce the overall duration of cardiopulmonary bypasses. Nevertheless, not all centers apply warmer temperatures with bilateral perfusion, as temperature management is frequently determined by the anticipated duration of circulatory arrest and surgical complexity rather than perfusion strategy alone. Temporal trends must also be taken into account, since more recent practice has progressively shifted toward warmer moderate hypothermic circulatory arrest [59], even in unilateral perfusion [60]. Importantly, a recent meta-analysis demonstrated that both randomized and propensity-matched data consistently associated deep hypothermic circulatory arrest with a higher incidence of stroke compared with moderate or mild hypothermia (*p* = 0.0029 and *p* = 0.019, respectively) during aortic arch procedures [61]. The analysis did not find a clear advantage of lower temperatures for either SCP technique.

In adjunct, while preoperative CT angiography can potentially identify high-risk patients [62], the vast majority of surgical centers primarily rely as intraoperative monitoring techniques on transcranial Doppler or near-infrared spectroscopy (NIRS) [63,64]. Conversion from unilateral to bilateral SACP is considered when unilateral cerebral oxygen saturation declines. Patients with a non-patent circle of Willis receiving (uSACP) have not demonstrated an increased incidence of neurological deficits [64], and several factors may contribute to this. A watershed infarct in the opposite cerebral hemisphere might present with varying clinical manifestations and may not always be promptly diagnosed, or collateral cerebral vessels may offer some contralateral perfusion in these patients [8].

There was contradictory evidence regarding the connection between unilateral and SACP and the duration of distal body circulatory arrest [7,8]. In the present analysis, no significant distinction was noted between the two perfusion strategies in this regard. However, a notable difference was identified concerning the cerebral perfusion time. This relation was confirmed at meta-regression, where longer cerebral perfusion, but not distal body circulatory arrest times, were associated with worse outcomes.

### Strengths and Limitations

While numerous comparable studies have explored variations in selective antegrade cerebral perfusion strategies during aortic arch surgery, our research investigated such outcomes for FET in ATAAD exclusively. This approach ensures that different techniques and aortic pathologies are not intermingled in our analysis.

The current meta-analysis is subject to certain limitations, though. Data was obtained mainly from retrospective studies, thus adding all related biases into the analysis. Moreover, due to the diversity in temperature measurement methods (rectal or nasopharyngeal) and cannulation strategies across the studies, we were unable to assess the correlation between these two parameters to the overall risk of neurological injury. Moreover, other missing parameters, like cerebral blood flow and mean perfusion pressure, could have impacted the outcomes.

Because all but one included study evaluated either unilateral or bilateral SACP exclusively within individual centers, observed differences between strategies may be confounded by center-specific expertise and practice patterns, limiting causal inferences about the effect of the SACP strategy itself.

## 5. Conclusions

Our analysis revealed consistent postoperative outcomes between uSACP and bSACP, despite significant different baseline patients’ features. This emphasizes the importance of tailoring the approach to the complexity of individual patient’s procedures, while a definitive superiority of bSACP over uSACP may be difficult to define. Of note, longer cerebral perfusion times, rather than distal body circulatory arrest durations, were associated with adverse postoperative outcomes. These findings collectively highlight the significance of considering the cerebral perfusion strategy according to the patient’s associated comorbidities to optimize outcomes during FET for ATAAD. Further studies, possibly randomized, are warranted to confirm these results.

## Figures and Tables

**Table 1:** Baseline patients’ characteristics of the included studies *.

Variable	Overall (N = 5983)	Unilateral (N = 3872)	Bilateral (N = 2111)	*p*-Value
Mean age, years	57.0 ± 6.9	51.6 ± 6.4	59.8 ± 5.4	**<0.001**
Male	75.8% (4538/5983)	79.3% (3069/3872)	69.6% (1469/2111)	**<0.001**
BMI	25.8 ± 1.8	25.5 ± 1.8	26.3 ± 1.8	0.1069
Connective tissue disease	4.8% (169/3556)	4.6% (112/2432)	5.1% (57/1124)	0.6014
Diabetes	5.6% (287/5147)	5.5% (197/3613)	5.9% (90/1534)	0.5986
COPD	2.9% (118/4138)	1.2%% (40/3239)	8.7% (78/899)	**<0.001**
History of stroke	5.2% (193/3733)	4.3% (128/2954)	8.3% (65/779)	**<0.001**
Hypertension	73.9% (3891/5265)	74.9% (2793/3730)	71.5% (1098/1535)	**0.0131**
CKD	5.2% (216/4169)	3.0% (98/3277)	13.2% (118/892)	**<0.001**
CAD	7.6% (309/4045)	6.7% (236/3498)	13.3% (73/547)	**<0.001**
Previous cardiac surgery	2.9% (110/3809)	2.5% (78/3124)	4.7% (32/685)	**0.003**
Acute neurological deficit	5.4% (192/3563)	1.7% (32/1871)	9.5% (160/1692)	**<0.001**
Hemodynamic compromise	6.0% (201/3358)	3.8% (110/2859)	18.2% (91/499)	**<0.001**
Hemopericardium	7.7% (301/3933)	5.2% (166/3182)	17.8% (135/751)	**<0.001**

* The denominator is based on the data availability among the included studies. Bold values mean *p* < 0.05. BMI = body mass index; CAD = coronary artery disease; CKD = chronic kidney disease; COPD = chronic obstructive pulmonary disease; CPR = cardiopulmonary resuscitation.

**Table 2:** Meta-analysis of the intraoperative data.

Outcome	Group	Studies	Total Pts	Estimate [95%CI]	Heterogeneity: I^2^, *p*-Value	Egger’s	Group Difference
Root replacement	Bilateral	26	1917	12.90% [7.77–20.66]	91.0%, *p* < 0.0001	*p* = 0.0012	***p* = 0.0012**
	Unilateral	13	3697	30.11% [23.95–37.07]	91.5%, *p* < 0.0001	*p* = 0.2448	
VSRR	Bilateral	20	1393	5.73% [2.94–10.88]	84.5%, *p* < 0.0001	*p* < 0.0001	*p* = 0.0833
	Unilateral	12	2541	2.09% [0.82–5.26]	80.5%, *p* < 0.0001	*p* = 0.5701	
Bentall	Bilateral	20	1393	8.64% [5.11–14.24]	78.9%, *p* < 0.0001	*p* = 0.0009	***p* < 0.0001**
	Unilateral	11	2508	28.64% [22.66–35.49]	85.5%, *p* < 0.0001	*p* = 0.0165	
Wheat	Bilateral	21	1608	1.36% [0.82–2.25]	0.0%, *p* = 0.9661	*p* = 0.0405	*p* = 0.6375
	Unilateral	11	2508	1.03% [0.36–2.92]	60.2%, *p* = 0.0051	*p* = 0.0028	
Root repair	Bilateral	23	1497	0.00% [0.00–0.00]	0.0%, *p* = 0.9964	*p* < 0.0001	*p* = 0.5901
	Unilateral	12	2541	1.76% [0.34–8.51]	87.8%, *p* < 0.0001	*p* = 0.1098	
AV repair	Bilateral	25	1778	1.70% [0.66–4.35]	84.0%, *p* < 0.0001	*p* < 0.0001	*p* = 0.4877
	Unilateral	12	2175	2.73% [1.06–6.87]	80.0%, *p* < 0.0001	*p* = 0.0008	
AV replacement	Bilateral	26	1917	5.20% [2.85–9.30]	81.6%, *p* < 0.0001	*p* = 0.0023	*p* = 0.0534
	Unilateral	12	2175	1.41% [0.42–4.58]	80.0%, *p* < 0.0001	*p* = 0.0039	
CABG	Bilateral	26	1917	7.64% [5.35–10.78]	64.8%, *p* < 0.0001	*p* = 0.0027	*p* = 0.5575
	Unilateral	13	3697	9.57% [4.84–18.02]	97.0%, *p* < 0.0001	*p* = 0.3827	
CPB time	Bilateral	29	2111	218.3 min [204.5–233.0]	99.0%, *p* < 0.0001	*p* = 0.0643	*p* = 0.9015
	Unilateral	14	2716	217.1 min [205.0–229.9]	98.2%, *p* < 0.0001	*p* = 0.4348	
CXC time	Bilateral	27	2081	125.0 min [116.1–134.5]	98.4%, *p* < 0.0001	*p* = 0.0937	*p* = 0.7091
	Unilateral	14	2716	122.4 min [112.7–132.9]	98.4%, *p* < 0.0001	*p* = 0.4867	
CA time	Bilateral	27	1965	38.9 min [32.2–47.0]	99.8%, *p* < 0.0001	*p* = 0.5360	*p* = 0.1019
	Unilateral	11	2129	30.7 min [24.9–37.9]	99.7%, *p* < 0.0001	*p* = 0.8003	
SACP time	Bilateral	19	1270	80.5 min [54.7–118.5]	99.9%, *p* < 0.0001	*p* = 0.0305	***p* = 0.0395**
	Unilateral	9	1994	49.8 min [39.0–63.5]	99.6%, *p* < 0.0001	-	
Target temperature	Bilateral	27	1984	26.0 °C [25.1–26.9]	99.8%, *p* < 0.001	*p* = 0.5198	***p* < 0.001**
	Unilateral	14	3828	22.5 °C [21.5–23.6]	99.9%, *p* < 0.001	*p* = 0.0375	
Stent length	Bilateral	18	852	9.99 cm [9.15–10.90]	100%, *p* < 0.0001	*p* = 0.9145	*p* = 0.7431
	Unilateral	5	200	10.25 cm [9.00–11.68]	100%, *p* < 0.0001	-	

Bold means *p* < 0.05. AV = aortic valve; CA = circulatory arrest; CABG = coronary artery disease; CI = confidence interval; CPB = cardiopulmonary bypass; CXC = cross-clamp; pts = patients; SACP = selective antegrade cerebral perfusion; VSRR = valve sparing root replacement.

**Table 3:** Meta-analysis of the postoperative outcomes.

Outcome	Group	No. of Studies	No. Total Pts	Estimate [95%CI]	Heterogeneity: I^2^, *p*-Value	Egger’s	Group Difference
Bleeding requiring surgical revision	Bilateral	19	1132	7.92% [5.14–12.02]	68.3%, *p* < 0.0001	*p* = 0.0009	*p* = 0.1494
	Unilateral	11	2501	4.64% [2.55–8.30]	76.0%, *p* < 0.0001	*p* = 0.1630	
Dialysis	Bilateral	15	1112	12.85% [8.59–18.78]	78.7%, *p* < 0.0001	*p* = 0.3028	*p* = 0.9939
	Unilateral	9	1955	12.82% [9.22–17.55]	57.1%, *p* = 0.0168	-	
CVA	Bilateral	22	1389	9.30% [6.93–12.38]	56.5%, *p* = 0.0006	*p* = 0.1377	*p* = 0.1932
	Unilateral	10	2057	6.37% [3.85–10.35]	59.3%, *p* = 0.0084	*p* = 0.3358	
SCI	Bilateral	25	1782	2.75% [1.77–4.26]	37.9%, *p* = 0.0296	*p* = 0.0017	*p* = 0.1525
	Unilateral	11	2136	4.34% [2.78–6.72]	37.6%, *p* = 0.0987	*p* = 0.7471	
Hospital mortality	Bilateral	27	1868	9.09% [7.42–11.10]	31.4%, *p* = 0.0619	*p* = 0.0840	*p* = 0.3483
	Unilateral	15	3872	7.87% [6.26–9.84]	55.0%, *p* = 0.0053	*p* = 0.8609	

CI = confidence interval; CVA = cerebrovascular accident; ICU = intensive care unit; pts = patients; SCI = spinal cord injury.

**Table 4:** Meta-regression of circulatory arrest and SACP times on mortality and neurological complications.

	Circulatory Arrest Time		SACP Time	
Outcome	OR (95%CI)	*p*-Value	OR (95%CI)	*p*-Value
Neurological complications				
Total population	1.01 (0.99–1.02)	0.3265	1.01 (1.00–1.02)	**0.0157**
uSACP	0.99 (0.90–1.08)	0.7942	1.01 (0.99–1.03)	0.0738
bSACP	1.01 (0.99–1.03)	0.2615	1.01 (0.99–1.02)	0.0928
Mortality				
Total population	1.00 (0.99–1.01)	0.5210	1.00 (0.99–1.01)	0.2315
uSACP	1.02 (0.99–1.05)	0.1491	1.01 (1.00–1.02)	**0.0473**
bSACP	1.00 (0.99–1.01)	0.9577	0.99 (0.99–1.01)	0.9067

Bold means *p* < 0.05. CI = confidence interval; OR = odds ratio.

## Data Availability

The datasets generated or analyzed during this study are available from the corresponding author upon reasonable request.

## Supplementary Materials

The following supporting information can be downloaded at: https://www.mdpi.com/article/10.3390/jcm14186392/s1, Table S1: Search strategy, Table S2: Outline of the included studies, Table S3: Distal anastomosis zone (Ishimaru) and epiaortic vessel management, Table S4: Risk of Bias in Non-Randomized Studies of Interventions (ROBINS-I) with traffic lights.

## Abbreviations

The following abbreviations are used in this manuscript:

ATAAD	Acute type A aortic dissection
CI	Confidence interval
CVA	Cerebrovascular accident
FET	Frozen elephant trunk
OR	Odds ratio
SACP	Selective antegrade cerebral perfusion
SCI	Spinal cord injury
TAR	Total arch replacement

## References

[1] Zhu Y., Lingala B., Baiocchi M., Tao J.J., Toro Arana V., Khoo J.W., Williams K.M., Traboulsi A.A.-R., Hammond H.C., Lee A.M. (2020). Type A Aortic Dissection-Experience Over 5 Decades: JACC Historical Breakthroughs in Perspective. J. Am. Coll. Cardiol..

[2] Sun L., Qi R., Zhu J., Liu Y., Zheng J. (2011). Total Arch Replacement Combined with Stented Elephant Trunk Implantation: A New “Standard” Therapy for Type a Dissection Involving Repair of the Aortic Arch?. Circulation.

[3] Kayali F., Jubouri M., Tan S.Z., Mohammed I., Bashir M. (2022). Aortic Remodeling in Aortic Dissection after Frozen Elephant Trunk: Overcoming the Challenges. J. Cardiovasc. Surg..

[4] Sá M.P., Jacquemyn X., Tasoudis P.T., Van den Eynde J., Erten O., Sicouri S., Dokollari A., Torregrossa G., Kurz S., Heuts S. (2022). Long-Term Outcomes of Total Arch Replacement versus Proximal Aortic Replacement in Acute Type A Aortic Dissection: Meta-Analysis of Kaplan-Meier-Derived Individual Patient Data. J. Card. Surg..

[5] Preventza O., Liao J.L., Olive J.K., Simpson K., Critsinelis A.C., Price M.D., Galati M., Cornwell L.D., Orozco-Sevilla V., Omer S. (2020). Neurologic Complications after the Frozen Elephant Trunk Procedure: A Meta-Analysis of More than 3000 Patients. J. Thorac. Cardiovasc. Surg..

[6] Hameed I., Rahouma M., Khan F.M., Wingo M., Demetres M., Tam D.Y., Lau C., Iannacone E.M., Di Franco A., Palaniappan A. (2020). Cerebral Protection Strategies in Aortic Arch Surgery: A Network Meta-Analysis. J. Thorac. Cardiovasc. Surg..

[7] Angeloni E., Melina G., Refice S.K., Roscitano A., Capuano F., Comito C., Sinatra R. (2015). Unilateral Versus Bilateral Antegrade Cerebral Protection During Aortic Surgery: An Updated Meta-Analysis. Ann. Thorac. Surg..

[8] Tian D.H., Wilson-Smith A., Koo S.K., Forrest P., Kiat H., Yan T.D. (2019). Unilateral Versus Bilateral Antegrade Cerebral Perfusion: A Meta-Analysis of Comparative Studies. Heart Lung Circ..

[9] Tasoudis P.T., Varvoglis D.N., Vitkos E., Ikonomidis J.S., Athanasiou T. (2023). Unilateral versus Bilateral Anterograde Cerebral Perfusion in Acute Type A Aortic Dissection Repair: A Systematic Review and Meta-Analysis. Perfusion.

[10] Naito N., Takagi H. (2025). Meta-Analysis: Bilateral and Unilateral Cerebral Perfusion in Type A Dissection. Thorac. Cardiovasc. Surg..

[11] Page M.J., McKenzie J.E., Bossuyt P.M., Boutron I., Hoffmann T.C., Mulrow C.D., Shamseer L., Tetzlaff J.M., Akl E.A., Brennan S.E. (2021). The PRISMA 2020 Statement: An Updated Guideline for Reporting Systematic Reviews. BMJ.

[12] Sterne J.A., Hernán M.A., Reeves B.C., Savović J., Berkman N.D., Viswanathan M., Henry D., Altman D.G., Ansari M.T., Boutron I. (2016). ROBINS-I: A Tool for Assessing Risk of Bias in Non-Randomised Studies of Interventions. BMJ.

[13] López Almodóvar L.F., Lima Cañadas P., Enríquez Puga A., Narváez Mayorga I., Buendía Miñano J.A., Sánchez Casado M., Cañas Cañas A. (2018). Single Low-Volume Center Experience with Frozen Elephant Trunk in Acute Type A Aortic Dissections. Aorta.

[14] Azuma S., Shimada R., Motohashi Y., Yoshii Y. (2023). Postoperative Results of the in Situ Fenestrated Open Stent Technique for Acute Aortic Dissection Type A. Gen. Thorac. Cardiovasc. Surg..

[15] Beckmann E., Martens A., Kaufeld T., Natanov R., Krueger H., Rudolph L., Haverich A., Shrestha M. (2022). Frozen Elephant Trunk in Acute Aortic Type a Dissection: Risk Analysis of Concomitant Root Replacement. Eur. J. Cardiothorac. Surg..

[16] Berger T., Kreibich M., Morlock J., Kondov S., Scheumann J., Kari F.A., Rylski B., Siepe M., Beyersdorf F., Czerny M. (2018). True-Lumen and False-Lumen Diameter Changes in the Downstream Aorta after Frozen Elephant Trunk Implantation. Eur. J. Cardiothorac. Surg..

[17] Berger T., Weiss G., Voetsch A., Arnold Z., Kreibich M., Rylski B., Krombholz-Reindl P., Winkler A., Mach M., Geisler D. (2019). Multicentre Experience with Two Frozen Elephant Trunk Prostheses in the Treatment of Acute Aortic Dissection†. Eur. J. Cardiothorac. Surg..

[18] Chen I.-M., Chen P.-L., Weng S.-H., Hsu C.-P., Shih C.-C., Chang H.-H., Wei J. (2018). Clinical Outcomes of VasoRing Connector in Patients with Acute Type A Aortic Dissection. Ann. Thorac. Surg..

[19] Chen X., Huang F., Xu M., Wang L., Jiang Y., Xiao L., Chen X., Qiu Z. (2010). The Stented Elephant Trunk Procedure Combined Total Arch Replacement for Debakey I Aortic Dissection: Operative Result and Follow-Up. Interact. Cardiovasc. Thorac. Surg..

[20] Chivasso P., Mastrogiovanni G., Bruno V.D., Miele M., Colombino M., Triggiani D., Cafarelli F., Leone R., Rosapepe F., De Martino M. (2022). Systematic Total Arch Replacement with Thoraflex Hybrid Graft in Acute Type A Aortic Dissection: A Single Centre Experience. Front. Cardiovasc. Med..

[21] Cuellar F.L., Oberhuber A., Martens S., Rukosujew A., Marchiori E., Ibrahim A. (2022). Analysis of Spinal Ischemia after Frozen Elephant Trunk for Acute Aortic Dissection: An Observational, Single-Center Study. Diagnostics.

[22] Cuko B., Pernot M., Busuttil O., Baudo M., Rosati F., Taymoor S., Modine T., Labrousse L. (2023). Frozen Elephant Trunk Technique for Aortic Arch Surgery: The Bordeaux University Hospital Experience with Thoraflex Hybrid Prosthesis. J. Cardiovasc. Surg..

[23] Dai L., Qiu J., Zhao R., Cao F., Qiu J., Wang D., Fan S., Xie E., Song J., Yu C. (2021). A Novel Sutureless Integrated Stented (SIS) Graft Prosthesis for Type A Aortic Dissection: A Pilot Study for a Prospective, Multicenter Clinical Trial. Front. Cardiovasc. Med..

[24] Fang C., Gao S., Ren X., Pang X., Zhao X., Ma Z., Wang C., Liu K. (2022). Comparison of Two Techniques in Proximal Anastomosis in Acute Type A Aortic Dissection. Front. Cardiovasc. Med..

[25] Goebel N., Nagib R., Salehi-Gilani S., Ahad S., Albert M., Ursulescu A., Franke U.F.W. (2018). One-Stage Hybrid Aortic Repair Using the Frozen Elephant Trunk in Acute DeBakey Type I Aortic Dissection. J. Thorac. Dis..

[26] Hohri Y., Yamasaki T., Matsuzaki Y., Hiramatsu T. (2020). Early and Mid-Term Outcome of Frozen Elephant Trunk Using Spinal Cord Protective Perfusion Strategy for Acute Type A Aortic Dissection. Gen. Thorac. Cardiovasc. Surg..

[27] Huang F., Li X., Zhang Z., Li C., Ren F. (2022). Comparison of Two Surgical Approaches for Acute Type A Aortic Dissection: Hybrid Debranching versus Total Arch Replacement. J. Cardiothorac. Surg..

[28] Iida Y., Fujii S., Shimizu H., Sawa S. (2019). Patterns of Aortic Remodelling after Total Arch Replacement with Frozen Elephant Trunk for Acute Aortic Dissection. Interact. Cardiovasc. Thorac. Surg..

[29] Iino K., Takago S., Saito N., Ueda H., Yamamoto Y., Kato H., Kimura K., Takemura H. (2022). Total Arch Replacement and Frozen Elephant Trunk for Acute Type A Aortic Dissection. J. Thorac. Cardiovasc. Surg..

[30] Inoue Y., Matsuda H., Omura A., Seike Y., Uehara K., Sasaki H., Kobayashi J. (2019). Comparative Study of the Frozen Elephant Trunk and Classical Elephant Trunk Techniques to Supplement Total Arch Replacement for Acute Type A Aortic Dissection†. Eur. J. Cardiothorac. Surg..

[31] Jakob H., Tsagakis K., Tossios P., Massoudy P., Thielmann M., Buck T., Eggebrecht H., Kamler M. (2008). Combining Classic Surgery with Descending Stent Grafting for Acute DeBakey Type I Dissection. Ann. Thorac. Surg..

[32] Kaneyuki D., Mogi K., Watanabe H., Otsu M., Sakurai M., Takahara Y. (2020). The Frozen Elephant Trunk Technique for Acute Retrograde Type A Aortic Dissection: Preliminary Results. Interact. Cardiovasc. Thorac. Surg..

[33] Katayama A., Uchida N., Katayama K., Arakawa M., Sueda T. (2015). The Frozen Elephant Trunk Technique for Acute Type A Aortic Dissection: Results from 15 Years of Experience†. Eur. J. Cardiothorac. Surg..

[34] Kong X., Ruan P., Yu J., Jiang H., Chu T., Ge J. (2022). Innominate Artery Direct Cannulation Provides Brain Protection during Total Arch Replacement for Acute Type A Aortic Dissection. J. Cardiothorac. Surg..

[35] Lin H., Chang Y., Guo H., Qian X., Sun X., Yu C. (2022). Prediction Nomogram for Postoperative 30-Day Mortality in Acute Type A Aortic Dissection Patients Receiving Total Aortic Arch Replacement with Frozen Elephant Trunk Technique. Front. Cardiovasc. Med..

[36] Liu H., Liu S., Zaki A., Wang X., Cong S., Yang Y., Li J., Lai H., Sun Y., Wei L. (2020). Quantifying the Learning Curve of Emergent Total Arch Replacement in Acute Type A Aortic Dissection. J. Thorac. Dis..

[37] Liu P., Wen B., Liu C., Xu H., Zhao G., Sun F., Zhang H., Yao X. (2021). En Bloc Arch Reconstruction with the Frozen Elephant Trunk Technique for Acute Type a Aortic Dissection. Front. Cardiovasc. Med..

[38] Liu Y., Jiang H., Wang B., Yang Z., Xia L., Wang H. (2022). Efficacy of Pump-Controlled Selective Antegrade Cerebral Perfusion in Total Arch Replacement: A Propensity-Matched Analysis. Front. Surg..

[39] Mariscalco G., Bilal H., Catarino P., Hadjinikolaou L., Kuduvalli M., Field M., Mascaro J., Oo A.Y., Quarto C., Kuo J. (2019). Reflection from UK Aortic Group: Frozen Elephant Trunk Technique as Optimal Solution in Type A Acute Aortic Dissection. Semin. Thorac. Cardiovasc. Surg..

[40] Morokuma H., Hamada K., Shimauchi K., Osaki J., Takahashi B., Yamamoto H., Hayashi N., Jinnouchi K., Itoh M., Yunoki J. (2023). How to Select the Optimal Size of Frozen Elephant Trunk in Total Arch Replacement for Type A Acute Aortic Dissection. Asian Cardiovasc. Thorac. Ann..

[41] Sato H., Fukada J., Tamiya Y., Mikami T. (2022). Morphometric Predictors of Aortic Remodeling after Frozen Elephant Trunk Repair of Type A Dissection. Ann. Vasc. Surg..

[42] Shen K., Tan L., Tang H., Zhou X., Xiao J., Xie D., Li J., Chen Y. (2022). Total Arch Replacement with Frozen Elephant Trunk Using a NEW “Brain-Heart-First” Strategy for Acute DeBakey Type I Aortic Dissection Can Be Performed Under Mild Hypothermia (≥30 °C) With Satisfactory Outcomes. Front. Cardiovasc. Med..

[43] Shi E., Gu T., Yu Y., Yu L., Wang C., Fang Q., Zhang Y. (2014). Early and Midterm Outcomes of Hemiarch Replacement Combined with Stented Elephant Trunk in the Management of Acute DeBakey Type I Aortic Dissection: Comparison with Total Arch Replacement. J. Thorac. Cardiovasc. Surg..

[44] Shi F., Wang Z. (2020). Acute Aortic Dissection Surgery: Hybrid Debranching Versus Total Arch Replacement. J. Cardiothorac. Vasc. Anesth..

[45] Shrestha M., Beckmann E., Krueger H., Fleissner F., Kaufeld T., Koigeldiyev N., Umminger J., Ius F., Haverich A., Martens A. (2015). The Elephant Trunk Is Freezing: The Hannover Experience. J. Thorac. Cardiovasc. Surg..

[46] Tochii M., Takami Y., Ishikawa H., Ishida M., Higuchi Y., Sakurai Y., Amano K., Takagi Y. (2019). Aortic Remodeling with Frozen Elephant Trunk Technique for Stanford Type A Aortic Dissection Using Japanese J-Graft Open Stent Graft. Heart Vessel..

[47] Wada T., Yamamoto H., Takagi D., Kadohama T., Yamaura G., Kiryu K., Igarashi I. (2022). Aortic Remodeling, Reintervention, and Survival after Zone 0 Arch Repair with Frozen Elephant Trunks for Acute Type A Aortic Dissection: Midterm Results. JTCVS Tech..

[48] Wang Z., Xue Y., Qian S., Liu Y., Zhu J., Sun L., Zhang H., Li H. (2023). Differences Between Sexes in Patients Who Underwent Total Arch Replacement and Frozen Elephant Trunk Procedures for Acute Dissection. Perfusion.

[49] Yamane Y., Uchida N., Mochizuki S., Furukawa T., Yamada K. (2017). Early- and Mid-Term Aortic Remodelling after the Frozen Elephant Trunk Technique for Retrograde Type A Acute Aortic Dissection Using the New Japanese J Graft Open Stent Graft. Interact. Cardiovasc. Thorac. Surg..

[50] Xiao Z., Meng W., Zhu D., Guo Y., Zhang E. (2014). Treatment Strategies for Left Subclavian Artery during Total Arch Replacement Combined with Stented Elephant Trunk Implantation. J. Thorac. Cardiovasc. Surg..

[51] Yang C., Hou P., Wang D., Wang Z., Duan W., Liu J., Yu S., Fu F., Jin Z. (2022). Serum Myoglobin is Associated with Postoperative Acute Kidney Injury in Stanford Type A Aortic Dissection. Front. Med..

[52] Yang S.-M., Xu P., Li C.-X., Huang Q., Gao H.-B., Li Z.-F., Chang Q. (2014). A Modified Total Arch Replacement Combined with a Stented Elephant Trunk Implantation for Acute Type A Dissection under Deep Hypothermic Circulatory Arrest and Selective Antegrade Cerebral Perfusion. J. Cardiothorac. Surg..

[53] Yoshitake A., Tochii M., Tokunaga C., Hayashi J., Takazawa A., Yamashita K., Chubachi F., Hori Y., Nakajima H., Iguchi A. (2020). Early and Long-Term Results of Total Arch Replacement with the Frozen Elephant Trunk Technique for Acute Type A Aortic Dissection. Eur. J. Cardiothorac. Surg..

[54] Zou Y., Teng P., Ma L. (2021). Four-Branched Graft Inversion Technique for the Distal Anastomosis in Acute Aortic Dissection. J. Cardiothorac. Surg..

[55] Okita Y. (2021). Frozen Elephant Trunk Usage in Acute Aortic Dissection. Asian Cardiovasc. Thorac. Ann..

[56] Baudo M., Rosati F., D’Alonzo M., Fiore A., Muneretto C., Benussi S., Di Bacco L. (2025). Total Arch Replacement with Ascyrus Medical Dissection Stent Versus Frozen Elephant Trunk in Acute Type A Aortic Dissection: A Meta-Analysis. J. Clin. Med..

[57] Yang B. (2021). Commentary: Individualize the Strategy of Cerebral Protection in Aortic Arch Surgery. JTCVS Tech..

[58] Misfeld M., Mohr F.W., Etz C.D. (2013). Best Strategy for Cerebral Protection in Arch Surgery—Antegrade Selective Cerebral Perfusion and Adequate Hypothermia. Ann. Cardiothorac. Surg..

[59] Czerny M., Grabenwöger M., Berger T., Aboyans V., Della Corte A., Chen E.P., Desai N.D., Dumfarth J., Elefteriades J.A., Etz C.D. (2024). EACTS/STS Guidelines for Diagnosing and Treating Acute and Chronic Syndromes of the Aortic Organ. Ann. Thorac. Surg..

[60] Tsai M.-T., Wu H.-Y., Hu Y.-N., Lin T.-W., Wen J.-S., Luo C.-Y., Roan J.-N. (2022). Safety Time and Optimal Temperature of Unilateral Antegrade Cerebral Perfusion in Acute Type A Aortic Dissection: A Single-Center 15-Year Experience. Acta Cardiol. Sin..

[61] Abjigitova D., Veen K.M., van Tussenbroek G., Mokhles M.M., Bekkers J.A., Takkenberg J.J.M., Bogers A.J.J.C. (2022). Cerebral Protection in Aortic Arch Surgery: Systematic Review and Meta-Analysis. Interact. Cardiovasc. Thorac. Surg..

[62] Papantchev V., Stoinova V., Aleksandrov A., Todorova-Papantcheva D., Hristov S., Petkov D., Nachev G., Ovtscharoff W. (2013). The Role of Willis Circle Variations during Unilateral Selective Cerebral Perfusion: A Study of 500 Circles. Eur. J. Cardiothorac. Surg..

[63] Harrer M., Waldenberger F.R., Weiss G., Folkmann S., Gorlitzer M., Moidl R., Grabenwoeger M. (2010). Aortic Arch Surgery Using Bilateral Antegrade Selective Cerebral Perfusion in Combination with Near-Infrared Spectroscopy. Eur. J. Cardiothorac. Surg..

[64] Urbanski P.P., Lenos A., Blume J.C., Ziegler V., Griewing B., Schmitt R., Diegeler A., Dinkel M. (2008). Does Anatomical Completeness of the Circle of Willis Correlate with Sufficient Cross-Perfusion during Unilateral Cerebral Perfusion?. Eur. J. Cardiothorac. Surg..

