# Supplementary material for: Unilateral vs. Bilateral Selective Cerebral Perfusion for Acute Type A Aortic Dissection with Frozen Elephant Trunk: Systematic Review and Meta-Analysis
Source: J Clin Med. 2025 Sep 10;14(18):6392. doi: 10.3390/jcm14186392 (PMC12470731; doi:10.3390/jcm14186392)
Supplement: Supplementary file 1 [file jcm-14-06392-s001.zip › jcm-3836189-supplementary.pdf]

Table S1 – Search strategy

#	Search
1	("cerebrally"[All Fields] OR "cerebrum"[MeSH Terms] OR "cerebrum"[All Fields] OR "cerebral"[All Fields] OR "brain"[MeSH Terms] OR "brain"[All Fields]) AND ("perfusable"[All Fields] OR "perfusate"[All Fields] OR "perfusates"[All Fields] OR "perfuse"[All Fields] OR "perfused"[All Fields] OR "perfuses"[All Fields] OR "perfusing"[All Fields] OR "perfusion"[MeSH Terms] OR "perfusion"[All Fields] OR "perfusions"[All Fields])
2	("unilateral"[All Fields] OR "unilaterally"[All Fields] OR "unilaterals"[All Fields])
3	("bilateral"[All Fields] OR "bilaterally"[All Fields] OR "bilaterals"[All Fields])
4	(1 AND 2) OR (1 AND 3)
5	"aorta, thoracic"[MeSH Terms] OR ("aorta"[All Fields] AND "thoracic"[All Fields]) OR "thoracic aorta"[All Fields] OR ("aortic"[All Fields] AND "arch"[All Fields]) OR "aortic arch"[All Fields]
6	"aortic dissection"[MeSH Terms] OR ("aortic"[All Fields] AND "dissection"[All Fields]) OR "aortic dissection"[All Fields]
7	("freezing"[MeSH Terms] OR "freezing"[All Fields] OR "frozen"[All Fields]) AND ("elephant s"[All Fields] OR "elephants"[MeSH Terms] OR "elephants"[All Fields] OR "elephant"[All Fields]) AND ("torso"[MeSH Terms] OR "torso"[All Fields] OR "trunk"[All Fields] OR "trunk s"[All Fields] OR "trunks"[All Fields])
8	5 AND 6 AND 7
9	4 AND 8

Table S2 – Outline of the included studies

Paper	Institution	Nation	Study period	Study	Prosthesis	Pts	SACP
Almodóvar 2019	Virgen de la Salud Hospital, Toledo	Spain	Jan 2011 - Dec 2016	OBS	E-Vita Open Plus	12	B
Azuma 2023	Kyoto Katsura Hospital, Kyoto	Japan	Sep 2014 - Mar 2022	OBS	J Graft Frozenix	67	B
Beckmann 2022	Hannover Medical School, Hannover	Germany	Mar 2013 - Dec 2019	OBS	Thoraflex Hybrid	115	B
Berger 2018	University Heart Center Freiburg University, Freiburg	Germany	Mar 2013 - May 2017	OBS	Thoraflex Hybrid	31	B
Berger 2019	University Hospital Salzburg; Hospital Hietzing, Vienna	Austria	Aug 2005 - Feb 2018	OBS	E-Vita Open	33	B
Chen 2010	Nanjing Cardiovascular Disease Research Institute, Nanjing First Hospital Affiliated to Nanjing Medical University, Nanjing	China	Aug 2004 - May 2009	OBS	Gianturco	28	B
Chen 2018	Taipei Veterans General Hospital, Taipei	Taiwan	Jun 2011 - Jul 2016	OBS	NR	33	U
Chivasso 2022	University Hospital San Giovanni di Dio e Ruggi d'Aragona, Salerno	Italy	Dec 2017 - Jan 2022	OBS	Thoraflex Hybrid	66	B
Cuellar 2022	University Hospital Muenster, Muenster	Germany	May 2015 - Apr 2019	OBS	Thoraflex Hybrid	31	B
Cuko 2023	Hopital Cardiologique de Haut-Leveque, Bordeaux University Hospital	France	Aug 2018 - Aug 2022	OBS	Thoraflex Hybrid	15	B/U
Dai 2022	Fuwai Hospital, National Center for Cardiovascular Diseases, Chinese Academy of Medical Sciences and Peking Union Medical College, Beijing	China	Aug 2020 - Sep 2020	OBS	Sutureless Integrated Stented graft	10	B
Fang 2022	Qilu Hospital of Shandong University, Jinan	China	Dec 2017 - May 2021	OBS	NR	143	B
Goebel 2018	Robert-Bosch-Hospital, Stuttgart	Germany	Oct 2009 - Dec 2016	OBS	E-Vita Open Plus	72	B

Hohri 2020	Japanese Red Cross Kyoto Daini Hospital, Kamigyo-ku, Kyoto	Japan	Jan 2013 - Dec 2017	OBS	J Graft Open Stent Graft, J Graft Frozenix	33	B
Huang F 2022	Fujian Provincial Hospital, Fuzhou	China	Jan 2017 - Dec 2019	OBS	Cronus	142	U
Iida 2019	Ogikubo Hospital, Tokyo	Japan	Apr 2015 - Nov 2018	OBS	J Graft Open Stent Graft	30	B
Iino 2022	Kanazawa University, Kanazawa	Japan	Mar 2016 - Feb 2020	OBS	J Graft Frozenix	50	B
Inoue 2019	National Cerebral and Cardiovascular Center, Suita	Japan	Jan 2012 - Feb 2018	OBS	J Graft Frozenix	33	U
Jakob 2008	West-German Heart Center Essen, University Hospital Essen, Essen	Germany	Jan 2001 - Oct 2007	OBS	Talent, E-vita Open	22	B
Kaneyuki 2020	Japanese Red Cross Narita Hospital,Narita-shi, Chiba; Funabashi Municipal Medical Center, Funabashi-shi, Chiba	Japan	Jan 2012 - Dec 2019	OBS	J Graft Open Stent Graft, J Graft Frozenix	32	B
Katayama 2014	Hiroshima City Asa Hospital, Hiroshima University, Hiroshima	Japan	1997 - 2012	OBS	NR	120	B
Kong 2022	The First Affiliated Hospital of USTC, University of Science and Technology of China (USTC), Hefei, Anhui	China	Oct 2020 - Nov 2021	OBS	Cronus	29	U
Lin 2022	Fuwai Hospital, Chinese Academy of Medical Sciences and Peking Union Medical College, Beijing	China	Jan 2010 - Dec 2020	OBS	Cronus	1156	U
Liu H 2020	Zhongshan Hospital, Shanghai Cardiovascular Institution, Fudan University, Shanghai	China	Jan 2013 - Dec 2016	OBS	Cronus	40	U
Liu P 2021	The First Affiliated Hospital of Zhengzhou University, Zhengzhou	China	Apr 2018 - Aug 2020	OBS	Cronus	44	U
Liu 2022	General Hospital of Northern Theater Command, Shenyang, Liaoning	China	Oct 2019 - Jul 2021	OBS	Cronus	192	B

Mariscalco 2019	Glenfield Hospital, University Hospitals of Leicester NHS Trust, Leicester; Manchester Royal Infirmary, Manchester; Papworth Hospital, Papworth; Liverpool Heart and Chest Hospital, Liverpool; University Hospital Birmingham NHS Foundation Trust, Birmingham; Barts Heart Center, St Bartholomew's Hospital, London; Royal Brompton Hospital, London; Derriford Hospital, Plymouth; University Hospital Southampton NHS Foundation Trust, Southampton	UK	Jun 2013 - Oct 2017	OBS	Thoraflex Hybrid	66	B
Morokuma 2023	Faculty of Medicine, Saga University, Saga	Japan	Jul 2014 - Jul 2022	OBS	J Graft Frozenix	44	B
Sato 2022	Otaru General Hospital, Otaru	Japan	Jan 2015 - Mar 2020	OBS	J Graft Frozenix	20	B
Shen 2022	The Second Xiangya Hospital of Central South University, Changsha	China	May 2017 - Dec 2020	OBS	Cronus	215	B
Shi 2014	First Affiliated Hospital, China Medical University, Shenyang	China	Jan 2006 - Dec 2011	OBS	Cronus	84	U
Shi 2020	Renmin Hospital of Wuhan University, Wuhan, Hubei	China	Jan 2017 - Jan 2019	PSM	NR	212	B
Shrestha 2015	Hannover Medical School, Hannover	Germany	Aug 2001 - Mar 2013	OBS	Chavan-Haverich, E-vita Open, Thoraflex	67	B
Sun 2011	Cardiovascular Institute and Fuwai Hospital, Peking Union Medical College, Chinese Academy of Medical Sciences, Beijing	China	Jan 2003 - Sep 2008	OBS	Cronus	148	U
Tochii 2019	Fujita Health University, Kutsukake, Toyoake, Aichi	Japan	Jan 2005 - Mar 2017	OBS	J Graft Open Stent Graft	22	B

Wada 2022	Akita University Graduate School of Medicine, Akita	Japan	Oct 2014 - Apr 2021	OBS	J Graft Frozenix	196	B
Wang Z 2022	Beijing Anzhen Hospital, Capital Medical University, Beijing	China	Jan 2009 - 2019	OBS	Cronus	1522	U
Xiao 2014	West China Hospital, Sichuan University Chengdu, Sichuan	China	Feb 2008 - Dec 2011	OBS	Cronus	33	U
Yamane 2017	Akane-Foundation Tsuchiya General Hospital, Hiroshima	Japan	Jan 2008 - Dec 2015	OBS	J Graft Open Stent Graft	24	U
Yang 2014	the Affiliated Hospital of Medical College, Qingdao University, Qingdao, Shandong	China	Feb 2008 - Feb 2013	OBS	Cronus	86	U
Yang C 2022	Xijing Hospital, Fourth Military Medical University, Xi'an	China	Dec 2017 - Jan 2020	OBS	Cronus	398	U
Yoshitake 2020	Saitama Medical University International Medical Center, Saitama	Japan	Jun 2007 - Dec 2018	PSM	UBE, Gianturco Z, TALENT, J Graft Open Stent Graft	139	B
Zou 2021	The First Affiliated Hospital of Medical College, Zhejiang University, Hang Zhou, Zhejiang Province	China	Jan 2017 - Jul 2019	OBS	Cronus	109	U

NR = not reported; OBS = observational study; PSM = propensity score matched study; Pts = patients; SACP = selective antegrade cerebral perfusion (B = bilateral; U = unilateral)

Table S3 – Distal anastomosis Zone (Ishimaru) and epiaortic vessel management

Paper	Epiaortic vessels	Distal anastomosis
Almodóvar 2019	Island reimplantation	Zone 2
Azuma 2023	Separate reimplantation	Zone 2
Beckmann 2022	Separate reimplantation	Zone 2 or Zone 3
Berger 2018	Separate reimplantation	Zone 2
Berger 2019	Both Island or separate reimplantation	Zone 2 n=2 (6%); Zone 3 n=31 (94%)
Chen 2010	Separate reimplantation	Zone 2
Chen 2018	Separate reimplantation	Distal arch
Chivasso 2022	Separate reimplantation	Usually Zone 2
Cuellar 2022	Separate reimplantation	Zone 3
Cuko 2023 uni	Separate reimplantation	NR
Cuko 2023 bi	Separate reimplantation	NR
Dai 2022	Separate reimplantation	Mostly Zone 2, some Zone 1
Fang 2022	Separate reimplantation	NR
Goebel 2018	Separate reimplantation	Zone 3
Hohri 2020	Separate reimplantation	Zone 2
Huang F 2022	Separate reimplantation	NR
Iida 2019	Separate reimplantation	Zone 2 n=15 (50%), Zone 3 n=15 (50%)
Iino 2022	Separate reimplantation	Zone 1: n=17, Zone 2: n=33
Inoue 2019	Separate reimplantation	Zone 3
Jakob 2008	NR	NR
Kaneyuki 2020	Separate reimplantation	Zone 1 or 2 n=23 (74%), Zone 3 n=9 (28%)
Katayama 2014	Separate reimplantation	Zone 2
Kong 2022	Separate reimplantation	Zone 2
Lin 2022	Separate reimplantation	Zone 2
Liu H 2020	Separate reimplantation	Zone 3
Liu P 2021	Island reimplantation	Zone 3
Liu 2022	Separate reimplantation	Distal arch

Mariscalco 2019	Separate reimplantation	Distal arch
Morokuma 2023	Separate reimplantation	Zone 1 n=4, Zone 2 n=33, Zone 3 n=7
Sato 2022	Separate reimplantation	Zone 1 n=11 (55.0%), Zone 2 n=9 (45.0%)
Shen 2022	Separate reimplantation	end of Zone 0 or beginning of Zone 1
Shi 2014	Separate reimplantation	Zone 2
Shi 2020	Separate reimplantation	Zone 2
Shrestha 2015	Separate reimplantation	Zone 2 or Zone 3
Sun 2011	Separate reimplantation	Zone 2
Tochii 2019	Separate reimplantation	Zone 3
Wada 2022	Separate reimplantation	Zone 0
Wang Z 2022	NR	NR
Xiao 2014	Separate reimplantation	Zone 2
Yamane 2017	Separate reimplantation	Zone 2
Yang 2014	Separate reimplantation	Zone 2
Yang C 2022	NR	NR
Yoshitake 2020	Separate reimplantation	Zone 2 or Zone 3
Zou 2021	Separate reimplantation	NR

Table S4 –Risk of Bias in Non-Randomized Studies of Interventions (ROBINS-I) with traffic lights

Study	D1	D2	D3	D4	D5	D6	D7	Overall
Almodóvar 2019	⊖	⊖	⊕	⊕	⊖	⊕	⊖	⊖
Azuma 2023	⊖	⊖	⊕	⊕	⊖	⊕	⊖	⊖
Beckmann 2022	⊗	⊖	⊕	⊕	⊖	⊕	⊖	⊗
Berger 2018	⊖	⊖	⊕	⊕	⊖	⊕	⊖	⊖
Berger 2019	⊖	⊖	⊕	⊕	⊖	⊕	⊖	⊖
Chen 2010	⊖	⊖	⊕	⊕	⊖	⊕	⊖	⊖
Chen 2018	⊗	⊖	⊕	⊕	⊖	⊕	⊖	⊗
Chivasso 2022	⊖	⊖	⊕	⊕	⊖	⊕	⊖	⊖
Cuellar 2022	⊖	⊖	⊕	⊕	⊖	⊕	⊖	⊖
Cuko 2023	⊖	⊕	⊕	⊕	⊖	⊕	⊖	⊖
Dai 2022	⊖	⊖	⊕	⊕	⊖	⊕	⊖	⊖
Fang 2022	⊗	⊖	⊕	⊕	⊖	⊕	⊖	⊗
Goebel 2018	⊖	⊖	⊕	⊕	⊖	⊕	⊖	⊖
Hohri 2020	⊖	⊖	⊕	⊕	⊖	⊕	⊖	⊖
Huang F 2022	⊗	⊖	⊕	⊕	⊖	⊕	⊖	⊗
Iida 2019	⊖	⊖	⊕	⊕	⊖	⊕	⊖	⊖
Iino 2022	⊖	⊖	⊕	⊕	⊖	⊕	⊖	⊖
Inoue 2019	⊗	⊖	⊕	⊕	⊖	⊕	⊖	⊗
Jakob 2008	⊖	⊖	⊕	⊕	⊖	⊕	⊖	⊖
Kaneyuki 2020	⊗	⊖	⊕	⊕	⊖	⊕	⊖	⊗
Katayama 2014	⊖	⊖	⊕	⊕	⊖	⊕	⊖	⊖
Kong 2022	⊖	⊖	⊕	⊕	⊖	⊕	⊖	⊖
Lin 2022	⊖	⊖	⊕	⊕	⊖	⊕	⊖	⊖
Liu H 2020	⊗	⊖	⊕	⊕	⊖	⊕	⊖	⊗
Liu P 2021	⊖	⊖	⊕	⊕	⊖	⊕	⊖	⊖
Liu 2022	⊕	⊖	⊕	⊕	⊖	⊕	⊖	⊖
Mariscalco 2019	⊖	⊖	⊕	⊕	⊖	⊕	⊖	⊖
Morokuma 2023	⊖	⊖	⊕	⊕	⊖	⊕	⊖	⊖
Sato 2022	⊖	⊖	⊕	⊕	⊖	⊕	⊖	⊖
Shen 2022	⊖	⊖	⊕	⊕	⊖	⊕	⊖	⊖
Shi 2014	⊗	⊖	⊕	⊕	⊖	⊕	⊖	⊗
Shi 2020	⊕	⊖	⊕	⊕	⊖	⊕	⊖	⊖
Shrestha 2015	⊗	⊖	⊕	⊕	⊖	⊕	⊖	⊗

Sun 2011	⊗	⊖	⊕	⊕	⊖	⊕	⊖	⊗
Tochii 2019	⊗	⊖	⊕	⊕	⊖	⊕	⊖	⊗
Wada 2022	⊖	⊖	⊕	⊕	⊖	⊕	⊖	⊖
Wang Z 2022	⊖	⊖	⊕	⊕	⊖	⊕	⊖	⊖
Xiao 2014	⊖	⊖	⊕	⊕	⊖	⊕	⊖	⊖
Yamane 2017	⊖	⊖	⊕	⊕	⊖	⊕	⊖	⊖
Yang 2014	⊖	⊖	⊕	⊕	⊖	⊕	⊖	⊖
Yang C 2022	⊖	⊖	⊕	⊕	⊖	⊕	⊖	⊖
Yoshitake 2020	⊕	⊖	⊕	⊕	⊖	⊕	⊖	⊖
Zou 2021	⊗	⊖	⊕	⊕	⊖	⊕	⊖	⊗

D1 = bias due to confounding; D2 = bias due to selection of participants; D3 = bias in classification of interventions; D4 = bias due to deviation from intended interventions; D5 = bias due to missing data; D6 = bias in measurements of outcomes; D7 = bias in selection of the reported results.
⊕ = low bias; ⊖ = moderate bias; ⊗ = serious bias.
